# Combining Biocontrol Agent With Plant Nutrients for Integrated Control of Tomato Early Blight Through the Modulation of Physio-Chemical Attributes and Key Antioxidants

**DOI:** 10.3389/fmicb.2022.807699

**Published:** 2022-03-23

**Authors:** Zoia Arshad Awan, Amna Shoaib, Muhammad Sarmad Iftikhar, Basit Latief Jan, Parvaiz Ahmad

**Affiliations:** ^1^Institute of Agricultural Sciences, University of the Punjab, Lahore, Pakistan; ^2^School of Agriculture and Food Sciences, University of Queensland, Brisbane, QLD, Australia; ^3^Department of Clinical Pharmacy, College of Pharmacy, King Saud University, Riyadh, Saudi Arabia; ^4^Botany and Microbiology Department, King Saud University, Riyadh, Saudi Arabia

**Keywords:** biocontrol, early blight, integrated disease management, tomato production, qPCR

## Abstract

Early blight (EB) is one of the major fungal diseases caused by *Alternaria solani* that is responsible for destructive tomato production around the globe. Biocontrol agent/s can be adequately implemented in an integrated management framework by using it in combination with vital plant nutrients, e.g., nitrogen, phosphorus, and potassium (NPK) and zinc (Zn). The current study was aimed to assess the integrated effect of a biocontrol agent *Bacillus subtilis* (BS-01) and the selective plant nutrients (NPK and Zn) on EB disease management and tomato crop performance. A field experiment was conducted for the off-season tomato production (under walk-in tunnels) in Punjab, Pakistan. The trial was set in a randomized complete block design (RCBD) and comprised nine treatments of a biocontrol agent (BS-01) either alone or in combination with the plant nutrients, *viz*., NPK (64:46:50 kg acre^–1^) and Zn (10 kg acre^–1^) as sustainable disease managing approach against EB. In addition, the biocontrol efficacy of *B. subtilis* (BS-01) on a fungal load of *A. solani* was estimated by quantitative PCR assays, where the foliar application of BS-01 on tomato plants either alone or in combination with the plant nutrients was done as a preventive measure. Our results revealed that the interactive effect of BS-01 with plant nutrients conferred significantly a varying degree of resilience in the infected tomato plants against EB by effectively modifying the content of total chlorophyll, carotenoids, and total phenolics along with the activities of antioxidant enzymes (SOD, CAT, POX, PPO, and PAL). In addition, the integrative effect of BS-01 and plant nutrients proved significantly effective in reducing pathogen load on inoculated tomato foliage, displaying the desired level of protection against *A. solani* infection. Besides, the complementary interaction of BS-01 + Zn + NPK worked synergistically to improve crop productivity by providing the highest marketable yield (21.61 tons acre^–1^) and net profit (361,363 Pakistani rupees acre^–1^). This integrated approach is put forward as a way to reduce the fungicide doses to control EB that would act as a sustainable plant protection strategy to generate profitable tomato production.

## Introduction

Tomato (*Solanum lycopersicum L.*) is an economically important fruity vegetable cultivated and consumed worldwide, because it is a rich source of vitamins (A, B, C, and K), minerals (Fe, P, K Ca, Zn, and Mg), sugars, organic acids, essential amino acids, dietary fibers, and antioxidants (Bauchet et al., 2017). Pakistan is one of the major producers of tomatoes, although its production and export are quite low annually due to the unavailability of high-yielding cultivars and the least resistance to biotic and abiotic constraints (Pervaiz et al., 2018). *Alternaria solani* is one of the most lethal tomato-infecting biotic restraints for its cultivation in the moist and warm tropical regions of the globe (Meitei et al., 2014). It causes economically important early blight (EB) disease that displayed >80% yield losses in Pakistan (Awan et al., 2019). For EB disease management, farmers mainly rely on agrochemicals (fungicides), but their undue usage and over-reliance have raised concerns for food safety, pathogen resistance, soil degradation, and environmental sustainability (Nashwa and Abo-elyousr, 2012; Adhikari et al., 2017). Therefore, there is a need to evolve crave alternative disease management approaches that would be supportive of sustainable agriculture without disturbing the environment. Bacteria-based biocontrol agents are powerful, bio-safer, and sustainable alternatives for profitable agricultural productivity, whereas specific strains of *Bacillus subtilis* have been proved effective in controlling many fungal diseases including EB disease because of the broad-spectrum activity of their antibiotics (Awan et al., 2019; Shoaib et al., 2019). Although plant disease resilience and tolerance are mainly related to genetics, they are affected by the environment and especially by mineral nutrients that are directly involved in plant defense as structural components (e.g., thickness of cell walls) and metabolic regulators (e.g., antioxidants, phytoalexins, and flavonoids (Dordas, 2008). Numerous studies have focused on the association between plant diseases and macronutrients, and ultimate attention has centered on the effects of nitrogen, phosphorus, and potassium (NPK) due to their deficiency in sundry soils and their elevated plant demand. These macronutrients play a chief role in the synthesis of several cellular constituents (DNA, RNA, and proteins), are responsible for the energy transfer, and contribute to various biochemical and physiological functions of a cell (Hasanuzzaman et al., 2018). Likewise, micronutrients, *viz*., Zn, B, Mn, Fe, and Cu, also take part in plant defense against several phytopathogens (Dordas, 2008), but zinc is predominantly documented for its major role in plant immune responses to several plant diseases (Khan et al., 2018). Although, as far as no general model has been proposed for Zn, this chief element involves several processes and is analyzed in different pathosystems (Awan et al., 2019). Likewise, rational amount of plant nutrients has been given subsequent attention toward disease control in a sustainable agriculture system to obtain the maximum yield (Shoaib and Awan, 2020). Henceforward, integrated disease management through plant nutrition along with a biological agent (BS-01) is an imperative and holistic approach that modulates as an eco-friendly and cost-effective strategy against tomato EB (Awan et al., 2019). The information regarding use of biocontrol (*B. subtilis*) and plant nutrients with peculiar emphasis to mitigate tomato EB is need to be addressed. This study was conducted to evaluate the impact of integrated microbial-mineral fertilization strategy using *B. subtilis* (BS-01) as a biocontrol agent and plant nutrients, *viz*., NPK and Zn-based mineral fertilizers in managing tomato EB in the field.

## Materials and Methods

### The Pathogen *Alternaria solani*

The fresh culture of *A. solani* (FCBP-1401) was procured from the First Fungal Culture Bank of Pakistan, isolated from the EB-infected tomato leaf and used for the preparation of conidial suspension (Awan et al., 2019). The fungal mycelium and conidia from 7-day-old pure fungal culture grown on synthetic potato dextrose agar (39 g L^–1^ of distilled water) were scrapped and washed off in autoclaved distilled water (Awan et al., 2018). The suspension was aseptically filtered twice to remove mycelia, and the pure conidial suspension was diluted to quantify for conidial count using a hemocytometer (Decelis et al., 2017).

### Biocontrol Agent *Bacillus subtilis* (BS-01)

Pure culture of *B. subtilis* (LC425129.1) was procured from the Environmental Plant Pathology Lab, isolated from the rhizospheric soil of the chickpea plant. On the basis of previous studies (Awan and Shoaib, 2019; Shoaib et al., 2019), BS-01 was used as a potential biocontrol agent. Microbial suspension of *B. subtilis* (BS-01) was prepared by harvesting bacterial cells in ice-cold 0.1 M phosphate-buffered saline (PBS; pH 6.8) from a 24-h-old pure bacterial culture grown in Luria–Bertani agar (1% tryptone, 1% NaCl 1%, 0.5% yeast extract 0.5% with pH 7.5).

### Experimental Site

A field experiment was conducted on the research land located at 01°30′15″N 74°18′23″ E, Faculty of Agricultural Sciences, University of the Punjab, Lahore, Pakistan. The same EB susceptible tomato genotype “Miracle” was used in the field experiment as used in previous studies (Awan et al., 2018, 2019; Awan and Shoaib, 2019; Shoaib et al., 2019; Shoaib and Awan, 2020). Likewise, on the basis of previous results of pot bioassays reported by Awan and Shoaib (2019), Shoaib et al. (2019), *B. subtilis* (BS-01) was selected along with the plant nutrients, *viz*., Zn (2X: 10 kg acre^–1^) and NPK (64:46:50 kg acre^–1^) to manage tomato EB in a susceptible tomato genotype *via* field trial. As the average temperature during December to February reaches 15°C ± 5°C, the trial was led in tunnels (walk-in) for the early growth and off-seasonal production of tomatoes.

### Experimental Design

Healthy tomato seeds of susceptible tomato genotype (Miracle) were sown in November for nursery preparation, tomato seedlings (20 days old) were transplanted after 3 weeks in soil beds of tunnels during mid of December (2017) and harvesting of tomato fruit was started after mid of March. Light irrigation was given immediately after seedling transplantation. Nine treatments were arranged in a randomized complete block design (RCBD) with two main subplots (*N* = 2; R_1_ and R_2_) and, in the main subplot (R_1_ and R_2_), each treatment consisted of further 10 replications (*n* = 10; r_1_–r_10_); hence, each treatment (Table 1) was exhibited into two main subplots with 10 replications.

**TABLE 1:** Treatments designed for a field experiment.

Treatments	
T_1_	−ve control (uninoculated)
T_2_	+ve control (inoculated with AS)
T_3_	BS-01 + AS
T_4_	Zn (2X) + AS
T_5_	BS-01 + Zn (2X) + AS
T_6_	NPK + AS
T_7_	BS-01 + NPK + AS
T_8_	Zn (2X) + NPK + AS
T_9_	BS-01 + Zn (2X) + NPK + AS

*BS-01, Bacillus subtilis; AS, Alternaria solani; NPK, nitrogen, phosphorus, and potassium; Zn, Zinc; −ve control, without inoculation of AS and application of nutrients; +ve control, with AS inoculation; Zn (2X: 10 kg acre^–1^) and NPK (64:46:50 kg acre^–1^).*

### Construction of Walk-in Tunnels for Field Trial

The total experimental area for the field trial was 38 m^2^ (6.55 × 5.81 m), the land was plowed with a rotavator, and the field’s soil was disinfected with a 2% formalin solution to eliminate soil infectious microorganisms. The soil was plowed again after a few days to remove residues of formalin. After plowing, the field was irrigated to maintain the field soil moisture (50–55%) for tomato seedlings. The field area was divided into two main plots (*N* = 2; R1 and R2) and 18 subplots (treatments) for the preparation of soil beds for tomato seedlings transplantation. Two main plots were separated for the construction of two walk-in tunnels (21 ft long and 10 ft wide). Tunnel-I has a plot area of 15.89 m^2^ (5.63 × 3.00 m), which was divided into eight subplots, whereas tunnel-II has a plot area of 19.65 m^2^ (6.55 × 3.00 m) and was divided into 10 subplots. Inter- and intra-distance between two subplots was maintained as 4 ft (1.22 m) and 1 ft (0.30 m), respectively, for cultural practices (hoeing and weeding). After blocking the field area, the tunnels were built by mild steel pipes (20–25 ft length, 40 mm diameter, and 1.6 mm thickness) and covered with plastic sheets (0.10 mm thick and 22 × 15 ft wide) in such a way that each tunnel was 7 ft high from the center and 4.5 ft high from both sides.

### Field Experiment

Twenty-day-old tomato seedlings were transplanted in soil, and plant-to-plant distance (0.152 m) and ridges to ridges distance (0.458 m) were maintained within a subplot of different treatments. As per treatment design (Table 1), the application of nutrients, biocontrol agent (BS-01), and pathogen were applied among the treatment as follows:

• For the treatments T_4_–T_9_, plant nutrients [NPK (64:46:50 kg acre^–1^) and Zn (2X: 10 kg acre^–1^)] were supplemented in the soil to the base of tomato plants after 15 days of seedling transplantation. Nitrogen fertilizer was applied in the form of urea (N: 46%) into two split doses according to the requirement of the tomato plant: the first was applied after 15 days of seedlings transplantation and the second dose after 45 days of seedlings transplantation at the flowering stage.
• After 10 days of nutrient application, tomato plants were sprayed with water only and were served as a negative control treatment (T_1_: without inoculation of AS and nutrient application). Whereas, plants were sprayed with 10–15 ml of *A. solani* conidial suspension (3.0 × 10^5^ conidia ml^–1^) served as the positive control (T_2_: inoculation with AS only).
• For the treatments T_2_, T_4_, T_6_, and T_8_, 10 days after nutrient application tomato, plants were primarily inoculated with 10–15 ml of *A. solani* conidial suspension (3.0 × 10^5^ conidia ml^–1^) through a hand sprayer.
• For the treatments, T_3_, T_5_, T_7_, and T_9_, 10 days after nutrient application, tomato plants were initially treated (sprayed) with 10–15 ml of microbial suspension (BS-01) prepared in PBS (OD595_nm_ = 2.0) (Abiodun et al., 2017); after 24 h, these treated plants were inoculated with 10–15 ml of conidial suspension.

The field soil was irrigated every 2–3 days at all stages of the tomato crop, and seasonal weeds were removed through digging. Plants were examined every 3–4 days after pathogen inoculation (DPI) to check the progression of EB symptoms.

### Assessments

#### Evaluation of Biocontrol Efficacy of *Bacillus subtilis* (BS-01) on the Pathogen Load

To assess the individual and integrative effect of *B. subtilis* (BS-01) as a preventive measure along with the plant nutrients on fungal load, the plants were harvested after 10 days of bacterial/pathogen inoculation (45-day-old plant). The reduction in fungal load due to the different treatments (T_1_–T_9_) was quantified by real-time quantitative PCR (qPCR) (Syed-Ab-Rahman et al., 2019).

##### DNA Extraction and Real-Time Quantitative PCR

DNA of the tomato leaves from the above-said treatments were isolated 2 weeks after pathogen/bacterial inoculation using cetyltrimethylammonium bromide protocol (Syed-Ab-Rahman et al., 2018). The extracted genomic DNA of tomato leaf was adjusted to 20 ng μl^–1^ using Thermo Scientific NanoDrop for qPCR amplification. For the pathogen quantification, a set of *A. solani* specific primers for cytochrome *b* and another set of primers for the plant’s housekeeping gene actin were used to amplify (Table 2).

**TABLE 2:** Primers to quantify fungal load (*Alternaria solani*) by qPCR.

Genes	Primers	Sequence of primers
Cytochrome *b*	As_Cytb_F	5′-TCA GGA ACT CTG TGG CGT ATC-3′
	As_Cytb_R	5′-TCA GAT GAA AGG GAG GGA GGA C-3
Actin	Act_F	5′-GGC AGG ATT TGC TGG TGA TGA TGC T-3′
	Act_R	5′-ATA CGC ATC CTT CTG TCC CAT TCC GA-3′

For qPCR, 10 μl of the reaction mixture was contained 5 μl of SYBR green, 0.7 μl of each primer (10 μM), and 3.6 μl of extracted DNA (20 ng ml^–1^). qPCR was performed using a CFX96 Touch™ Real-Time PCR detection system (Life Science Research, Bio-Rad). Thermal cycling conditions were set as follows: initial denaturation for 2 min at 95°C, followed by for 5 s at 95°C and 10 s at 60°C for 45 cycles, and a final extension step at 60°C and 95°C for 5 s. Results were analyzed by the inbuilt software (CFX Manager™ software) system connected to the CFX96 Touch™ Real-Time PCR detection system.

#### Physio-Chemical Attributes

Tomato leaves were sampled (10 samples replicate^–1^) from each treatment to assess the physiological and biochemical changes [photosynthetic pigments, i.e., total chlorophyll contents and carotenoids (CARO), total phenolic content, total protein content (TPC), and defensive antioxidant enzymes, *viz*., superoxide dismutase (SOD), catalase (CAT), peroxidase (POX), polyphenol oxidase (PPO), and phenylalanine ammonia-lyase activity (PAL) in tomato plant under EB stress]. For this assessment, leaves were sampled twice at 10 DPI (45-day-old plant) and 30 DPI (65-day-old plant) following similar protocols as described by Awan and Shoaib (2019).

#### Disease Attributes

The data regarded disease intensity in terms of disease incidence (DI) and percent severity index (PSI) were recorded twice after 20 DPI (45-day-old plant) and 50 DPI (75-day-old plant) using the formula mentioned by Awan et al. (2018). Disease severity was measured using a disease rating scale mentioned in Table 3 (Pandey et al., 2003) for PSI.

**TABLE 3:** The disease rating scale for tomato early blight (Pandey et al., 2003).

Scale	Description
0	Leaves free from infection
1	<5% of leaf area affected by small irregular spots
2	5.1–10% of foliage covered with small irregular brown spots with concentric rings
3	10.1–25% of leaf area covered by irregular brown spots with concentric rings and enlarged lesions
4	25.1–50% area of the leaf-covered with coalesced lesions to form irregular and appears as a typical blight symptom
5	>50% of leaf area covered with lesions coalesce to form irregular and appears as a typical blight symptom

#### Agronomical and Growth Attributes

Agronomic traits such as the total number of branches, flowering branches, fruiting branches, an ad flowers and fruits per plant were carefully monitored after 35 DPI (70-day-old plant) and were reckoned at 30 DPI (60-day-old plant), and data were recorded.

Growth attributes related to shoot and root parameters of tomato plant [length and weight (fresh and dry)] were recorded at 60 DPI (90-day-old plant) following the protocol of Awan et al. (2018, 2019).

#### Yield Attributes

##### Fruit Grading and Yield

Harvesting of tomato fruits was started from 90-day-old plant after tomato seedlings transplantation when fruits are riped for first picking. Later tomatoes were regularly harvested after every 2–3 days until the end of the last harvest (160-day-old plant).

Yield attributes included quality and quantity of tomato fruits, where the quality of tomatoes was graded on the basis of the size of tomato (diameter), *viz*., “A” grade: above 3.5 cm diameter, “B” grade: 2.5–3.5 cm diameter, and “C” grade: below 2 cm diameter. As far as the quantitative attribute of tomato yield was concerned, each harvest of tomatoes was weighed, and recorded data were used to calculate the yield (kilogram) per treatment. Finally, the data were used to calculate the marketable yield, unmarketable yield, and total tomato yield (ton acre^–1^).

##### Economic Analysis

Economic analysis was estimated from the price of marketable yield [Pakistani rupee (PKR) acre^–1^]. The total revenue (TR), total cost (TC), net return (NR) in PKR, and benefit-cost ratio (B:C) of tomato production were calculated by using the formula employed by Liu et al. (2015) and Imran et al. (2018).

TR = Q × P [Q = yield (kg acre^–1^), P = price of yield (PKR)]
TC = V × X [V = input prices, X = input purchase quantity]
NR = TR-TC (PKRs acre^–1^)
B:C = TR/TC

### Statistical Analysis

Statistix 8.1 (software) was used to analyze the data recorded from the field experiment, and data were normalized by the Kolmogorov–Smirnov test initially and submitted to ANOVA indicated a significant difference among the treatments when their means were compared using Fisher’s least significant difference (LSD) test at 95% confidence level. Pearson correlation was also calculated to assess the relationships among the different physio-chemical attributes.

The clustering and ordination were carried out in RStudio, and classification enhanced biplot was constructed to understand the behavior of the physio-chemical, disease, agronomic, growth, and yield attributes under different treatments. Data were first standardized, and average squared Euclidean distance (SED) was calculated. A simple matching coefficient (SMC) was calculated by taking away SED from one. SMC was compulsory to use in classification enhanced biplot to understand the similarity and differences among different treatments in a field trial. In addition, data were first scaled and were used as a matrix to construct the heatmap that was created through RStudio using the R package “d3heatmap” reference. Dendrograms were optimized as in classification. The color scale was used from blue to yellow and red for the highest to lowest values, respectively.

## Results

### Evaluation of Control Efficacy Treatments on the Pathogen Load

**FIGURE 1:**
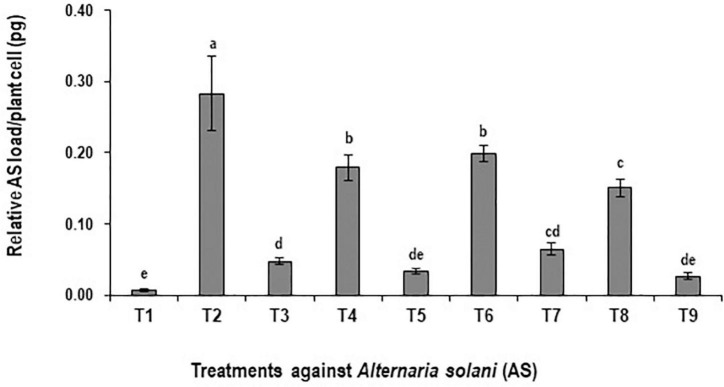
Effect of *Bacillus subtilis* (BS-01) on the relative fungal load of *Alternaria solani* (AS) in tomato foliage using qPCR after 10 days of pathogen inoculation. Values with different letters show a significant difference (*p* ≤ 0.05) in the mean value of replicates as determined by the LSD test. Error bars indicate the standard errors of the mean of replicates. −ve control, without inoculation of AS and nutrient application; +ve control, with inoculation of AS only (foliar application). T_1_, −ve control; T_2_, +ve control; T_3_, BS-01 + AS; T_4_:, Zn (2X) + AS; T_5_, BS-01 + Zn (2X) + AS; T_6_, NPK + AS; T_7_, BS-01 + NPK + AS; T_8_, Zn (2X) + NPK; and T_9_, BS-01 + Zn (2X) + NPK + AS.

The preventive effects of BS-01 application alone and in combination with plant nutrients were evaluated by quantifying pathogen load (*A. solani*) in tomato foliage using a set of fungal specific primers “cytochrome *b*”. The significantly high fungal load of *A. solani* by 98% (at *p* ≤ 0.05) was quantified in the positive control (plants inoculated with *A. solani*) as compared to the negative control (healthy plants without pathogen inoculation) (Figure 1), where the maximum fungal genomic DNA as 0.283 pg was detected in the infected tomato leaves. Among the disease control treatments such as T_9_: BS + AS + NPK + Zn (2X) followed by T_5_: BS + AS + Zn (2X) and T_3_: BS + AS exhibited a significant reduction in pathogen load by 83–90% as compared to the positive control treatment. Hence, the relative fungal load reduced significantly by ∼90% under the potential effect of BS-01 and plant nutrients (T_9_, T_5_, and T_3_) against tomato EB (Figure 1). However, plants raised without biocontrol agent (BS-01) in treatments T_4_: AS + Zn (2X), T_6_: AS + NPK, and T_8_: AS + NPK + Zn (2X) also significantly reduced the pathogen load but to some extent (30–47%) over the positive control.

### Physio-Chemical Attributes

Generally, the applications of BS-01 in combination with plant nutrients [Zn (2X: 10 kg acre^–1^) and NPK (64:46:50 kg acre^–1^)] improved physio-chemical traits of tomato plants under EB stress in the field.

#### Total Chlorophyll Content and Carotenoids

*Alternaria solani* infected plants in positive control treatment were significantly reduced total chlorophyll content (TCC) by ∼55% (*p* ≤ 0.05) and recorded 6.1 and 7.2 mg g^–1^ of fresh weight of leaf at 10 and 30 DPI, respectively, as compared to the healthy plants in the negative control (14.4 and 15.2 mg g^–1^ of fresh weight leaf at10 and 30 DPI, respectively). The highest TCC (∼22.0 mg g^–1^) was recorded with the application of BS-01 + Zn (2X) + NPK in T_9_ at both 10 and 30 DPI; it increased three- to fourfold over positive control and almost equal (∼20.0 mg g^–1^) with the application BS-01 + NPK. The rest of the treatments also enhanced the said attribute significantly (*p* ≤ 0.05) up to 2.0- to 2.5-fold as compared to the positive control (Table 4).

**TABLE 4:** Effect of *Bacillus subtilis* (BS-01) and mineral nutrients [Zn (2X: 10 kg acre^–1^) and NPK (64:46:50 kg acre^–1^)] on the physio-chemical attributes in tomato plants inoculated with *Alternaria solani* (AS) in the field after 10 and 30 days of pathogen inoculation (DPI).

Parameters	DPI	T_1_	T_2_	T_3_	T_4_	T_5_	T_6_	T_7_	T_8_	T_9_
Total chlorophyll content	10 DPI	14.4^cd^	6.1^e^	11.7^d^	12.7^d^	15.2^cd^	14.8^cd^	19.7^ab^	16.5^bc^	21.1^a^
(mg g^–1^)	30 DPI	15.2^cd^	7.2^e^	12.2^d^	13.4^d^	16.0^cd^	15.6^cd^	20.7^ab^	17.4^bc^	22.8^a^
Carotenoids	10 DPI	11^de^	6.5^f^	9.9^e^	10.5^e^	12.8^bc^	11.8^cd^	13.5^ab^	12.4^bc^	14.8^a^
(mg g^–1^)	30 DPI	14.8^d^	5.7^f^	12.4^e^	13.6^de^	17.0^bc^	15.3^cd^	18.0^b^	16.7^bc^	19.9^a^
Total phenolic content	10 DPI	40^f^	61^e^	166^cd^	147^d^	183^bc^	163^cd^	195^ab^	181^bc^	206^a^
(mg g^–1^)	30 DPI	43^e^	57^e^	189^cd^	168^d^	218^ab^	187^cd^	220^ab^	208^bc^	236^a^
Total protein content	10 DPI	0.66^f^	1.19^e^	2.09^cd^	2.07^d^	2.64^b^	2.23^cd^	2.72^ab^	2.35^c^	2.94^a^
(mg g^–1^)	30 DPI	0.86^f^	1.28^e^	2.35^cd^	2.33^d^	2.93^b^	2.51^cd^	3.01^ab^	2.63^c^	3.25^a^
Superoxide dismutase	10 DPI	2.04^d^	1.67^d^	2.81^bc^	2.65^c^	3.05^a–c^	2.67^c^	3.25^ab^	2.91^a–c^	3.34^a^
(units min^–1^ mg^–1^ of protein)	30 DPI	2.04^d^	1.64^d^	2.79^bc^	2.63^c^	3.04^a–c^	2.66^c^	3.23^ab^	2.90^a–c^	3.34^a^
Catalase	10 DPI	0.21^b–d^	0.09^e^	0.20^cd^	0.20^cd^	0.24^b^	0.18^d^	0.30^a^	0.24^bc^	0.29^a^
(units min^–1^ mg^–1^ of protein)	30 DPI	0.18^d^	0.09^e^	0.19^cd^	0.19^cd^	0.24^b^	0.17^d^	0.29^a^	0.23^bc^	0.29^a^
Peroxidase	10 DPI	19^d^	20^d^	37^bc^	33^c^	36^bc^	33^c^	40^ab^	36^bc^	43^a^
(units min^–1^ mg^–1^ of protein)	30 DPI	16^c^	18^c^	34^b^	31^b^	35^b^	32^b^	36^b^	34^b^	42^a^
Polyphenol oxidase	10 DPI	1.83^ab^	0.59^e^	1.35^cd^	1.05^d^	1.63^a–c^	1.09^d^	1.53^bc^	1.42^c^	1.91^a^
(units min^–1^ mg^–1^ of protein)	30 DPI	1.52^ab^	0.68^d^	1.26^bc^	0.98^c^	1.54^ab^	1.02^c^	1.45^b^	1.33^b^	1.82^a^
Phenylalanine ammonia-lyase	10 DPI	0.60^d^	0.80^d^	1.70^bc^	1.74^bc^	1.95^ab^	1.48^c^	2.03^ab^	1.93^ab^	2.14^a^
(units min^–1^ mg^–1^ of protein)	30 DPI	0.54^e^	0.78^e^	1.78^cd^	1.81^b–d^	2.06^a–c^	1.55^d^	2.16^ab^	2.04^a–c^	2.28^a^

*Values with different letters show a significant difference (p ≤ 0.05) in the mean value of replicates as determined by the LSD test. −ve control, without inoculation of AS and nutrient application; + ve control, with inoculation of AS only (foliar application); T_1_, −ve control; T_2_, +ve control; T_3_, BS-01 + AS; T_4_, Zn (2X) + AS; T_5_, BS-01 + Zn (2X) + AS; T_6_, NPK + AS; T_7_, BS-01 + NPK + AS; T_8_, Zn (2X) + NPK; and T_9_, BS-01 + Zn (2X) + NPK + AS.*

Leaf CARO were decreased significantly by 40% (*p* ≤ 0.05) at 10 DPI but more drastically by 62% at 30 DPI in positive control over the negative control. Like TCC, CARO was significantly increased (*p* ≤ 0.05) by two- to threefold in a combination treatment T_9_: BS-01 + Zn (2X) + NPK followed by combination treatments BS-01 + NPK (T_7_), BS-01 + Zn (2X) (T_5_), Zn (2X) + NPK (T_8_), and NPK only (T_6_) at both DPI. The application of BS-01 and Zn (2X) alone also showed enhancement in the CARO by 55% at 10 DPI and 130% at 30 DPI over positive control (Table 4).

#### Total Phenolic Content

Total phenolic content (PHE) of *A. solani* infected plants in the positive control (61 mg g^–1^ of fresh weight of leaf at both DPI) was increased significantly by 55% (*p* ≤ 0.05) concerning a negative control treatment. All other combination treatments (T_4_–T_9_) induced significant (*p* ≤ 0.05) production of the phenolic content over positive control. Likewise, PHE production increased tremendously up to four- to fivefold in combination treatment (T_9_) BS-01 + Zn (2X) + NPK followed by in all other combination treatments (T_8_, T_7_, and T_5_), *viz*., Zn (2X) + NPK, BS-01 + NPK, and BS-01 + Zn (2X) by three- to fourfold, as well as threefold increased in a single treatment application of BS-01 or nutrients (Zn or NPK) (Table 4).

#### Total Protein Content

Total protein content was also significantly increased in all treatments. However, the AS-inoculated leaf in positive control displayed an improvement of 81% at 10 DPI and 49% at 30 DPI over the negative control. BS-01 in combination with Zn (2X) and NPK in treatment T_9_ showed a significant increase in TPC by two- to threefold (*p* ≤ 0.05), followed by the application treatments BS-01 + NPK and BS-01 + Zn (2X) at both DPI. TPC also improved in the rest of the treatments by 70–100% at both DPI over positive control (Table 4).

#### Activities of Defensive Enzymes

The activity of SOD has not affected (*p* ≥ 0.05) the EB stressed plant in positive control as compared to the healthy plants in negative control at both DPI. However, the activity of defensive enzymes was raised significantly up to twofold (*p* ≤ 0.05) in a combination of BS-01 with Zn (2X) and NPK and almost equal with the other combination treatments (T_5_–T_8_) at both DPI. Hence, applied treatments, *viz*., BS-01 + NPK, BS-01 + Zn (2X), and Zn (2X) + NPK, showed significant elevation in SOD up to five-, four-, and threefold (*p* ≤ 0.05), respectively, at both DPI over positive control. The rest of the treatments of solitary application of BS-01, Zn (2X), and NPK showed a 60–70% elevation in the activity of enzymes over positive control (Table 4).

The CAT activity was significantly reduced by 40–50% (*p* ≤ 0.05) in a pathogen-inoculated plant (positive control) as compared to the healthy plant in the negative control. A combination treatment of BS-01 + Zn (2X) + NPK, as well as combination treatments of BS-01 + NPK and BS-01 + Zn (2X), showed significant highest (*p* ≤ 0.05) activity of CAT (threefold) at both DPI over positive control. Whereas, the remaining treatments, including the solitary application of BS-01, Zn, and NPK, displayed a statistically similar increase of twofold in CAT activity at both DPI over positive control (Table 4).

The POX activity was not affected (*p* ≥ 0.05) in the positive control as compared to the negative control at both DPI. Like the activities of former enzymes, a combination treatment (T_9_) of BS-01 + Zn (2X) + NPK induced a significant increase (*p* ≤ 0.05) by 155% and 134% at 10 and 30 DPI, respectively, over positive control. The rest of the treatments (T_3_–T_8_) exhibited a significant elevation of 65–100% at 10 DPI and 75–100% at 30 DPI over positive control (Table 4).

The activity of PPO decreased significantly (*p* ≤ 0.05) by 68% at 10 DPI and by 55% at 30 DPI in AS-inoculated plants as compared to the healthy plants. The activity of the PPO enzyme was significantly enhanced (*p* ≤ 0.05) up to two- to threefold in treatments BS-01 + Zn (2X) + NPK and BS-01 + Zn (2X) at both DPI over positive control. Combination treatments of BS-01 + NPK and Zn (2X) + NPK also significantly improved enzyme activities by 100–150% (*p* ≤ 0.05) at both DPI over positive control. Solitary application of BS-01 increased PPO activity by 128% at 10 DPI and 86% at 30 DPI, whereas the application of nutrients Zn (2X) or NPK alone enhanced PPO 82% at 10 DPI and 47% at 30 DPI, respectively, over positive control (Table 4).

The activity of PAL (defense-related enzyme) was not affected (*p* ≥ 0.05) in pathogenic infected plants in positive control over negative control at both DPI. The activity of PAL increased significantly (*p* ≤ 0.05) up to threefold in a combination treatment BS-01 + Zn (2X) + NPK followed by other combination treatments, *viz*., BS-01 + Zn, BS-01 + NPK, and Zn (2X) + NPK, by two- to threefold at both DPI. Solitary application of BS-01, Zn (2X), and NPK showed a similar increase in the activity of PAL by twofold at both DPI over positive control (Table 4).

#### Correlation Matrix Among Physiological Attributes

**FIGURE 2:**
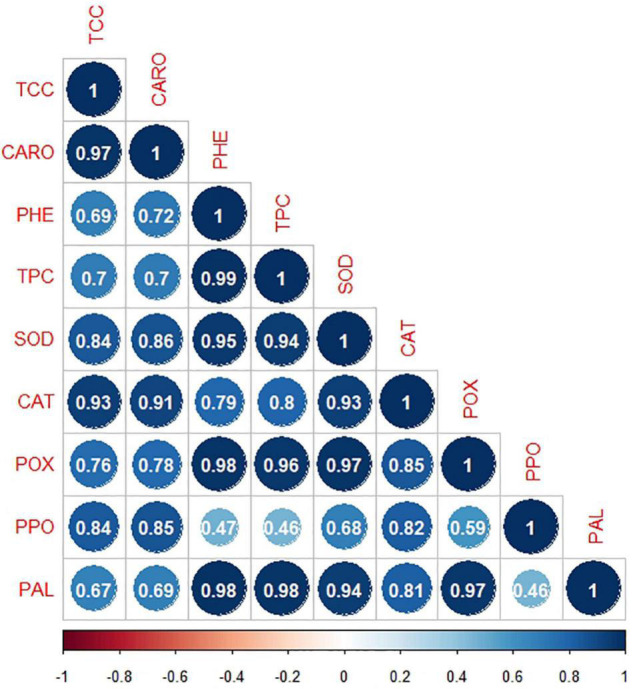
Correlation matrix of physio-chemical attributes (TCC, total chlorophyll content; CARO, carotenoids; PHE, total phenolic content; TPC, total protein content; SOD, superoxide dismutase; CAT, catalase; POX, peroxidase; PPO, polyphenol oxidase; PAL, phenylalanine ammonia-lyase) under the effect of *Bacillus subtilis* (BS-01) and mineral nutrients [Zn (2X: 10 kg acre^–1^) and NPK (64:46:50 kg acre^–1^)] in tomato plants inoculated with *Alternaria solani* (AS) in the field after 10 and 30 days of pathogen inoculation (DPI). strong association, *r* = 0.46–0.59 significant at *p* ≤ 0.05; moderately Strong association, *r* = 0.65–0.8 significant at *p* ≤ 0.01; and very strong association, *r* = 0.81–1.00 significant at *p* ≤ 0.001.

All physiological attributes showed moderately strong (*r* = 0.46–0.59; *p* ≤ 0.05), strong (*r* = 0.65–0.8; *p* ≤ 0.01), and very strong association (*r* = 0.81–1.00; *p* ≤ 0.001) with each other. Both photosynthetic pigments (TCC and CARO) were positively and significantly correlated (*r* = 0.97; *p* ≤ 0.001) with each other. PHE exhibited positive and significant associations (*r* = 0.95–0.98; *p* ≤ 0.001) with TPC, SOD, POX, and PAL. Likewise, TPC displayed positive and significant correlation (*r* = 0.80–0.98) with SOD, CAT, POX, and PAL at *p* ≤ 0.01 and *p* ≤ 0.001. Furthermore, all defense-related enzymes (SOD, CAT, POX, PPO, and PAL) showed significant correlation (*p* ≤ 0.01 and *p* ≤ 0.001) with each other. The activity of SOD exhibited strong association (*r* = 0.93–0,97; *p* ≤ 0.001) with CAT, POX, and PAL enzymes. PAL as a defensive enzyme had significantly strong association (*r* = 0.98; *p* ≤ 0.001) with PHE, TPC, SOD, CAT, and POX (Figure 2).

### Disease Assessment

Tomato plants inoculated with pathogen only (positive control) had a greatest disease severity (DI: 90%; PSI: 75%) at 20 days (DI: 100%; PSI: 85%) and 50 DPI. All treatments reduced EB significantly but the combination of BS-01 + Zn (2X) + NPK showed a significantly highest reduction in disease severity (DI: 25%; PSI: 30%) (*p* ≤ 0.05) at 20 and 50 DPI. In addition, the combined applications of BS-01 with Zn or NPK displayed a reduction in DI by 30–40% and PSI by 35–38% (*p* ≤ 0.05) at both DPI (20 and 50) (Table 5).

**TABLE 5:** Effect of *Bacillus subtilis* (BS-01) and mineral nutrients [Zn (2X: 10 kg acre^–1^) and NPK (64:46:50 kg acre^–1^)] on disease in tomato plants inoculated with *Alternaria solani* (AS) in the field after 20 and 50 days of pathogen inoculation (DPI).

Treatments		Days after pathogen inoculation (DPI)			
		20 DPI		50 DPI	
		Disease incidence (%)	Percent severity index (%)	Disease incidence (%)	Percent severity index (%)
T_1_	−ve control	00.00^e^	00.00^g^	00.00^g^	00.00^g^
T_2_	+ve control (*A. solani*)	90.21^a^	85.21^a^	100.00^a^	85.43^a^
T_3_	BS-01 + AS	50.44^c^	45.19^d^	50.34^d^	45.26^cd^
T_4_	Zn (2X) + AS	55.12^bc^	53.36^c^	60.23^c^	53.39^bc^
T_5_	BS + Zn (2X) + AS	30.34^d^	35.15^e^	35.17^ef^	35.33^e^
T_6_	NPK + AS	65.11^b^	62.22^b^	70.39^b^	62.21^b^
T_7_	BS-01 + NPK + AS	35.01^d^	38.11^de^	40.41^e^	38.17^de^
T_8_	Zn (2X) + NPK + AS	50.13^c^	42.81^d^	60.32^c^	43.48^de^
T_9_	BS-01 + Zn (2X) + NPK + AS	25.18^d^	30.44^f^	30.43^f^	30.16^f^

*Values with different superscript letters in columns show a significant difference (p ≤ 0.05) in the mean value of replicates as determined by the LSD test. −ve control, without inoculation of AS and nutrient application; +ve control, with inoculation of AS only (foliar application).*

### Agronomic Traits

**FIGURE 3:**
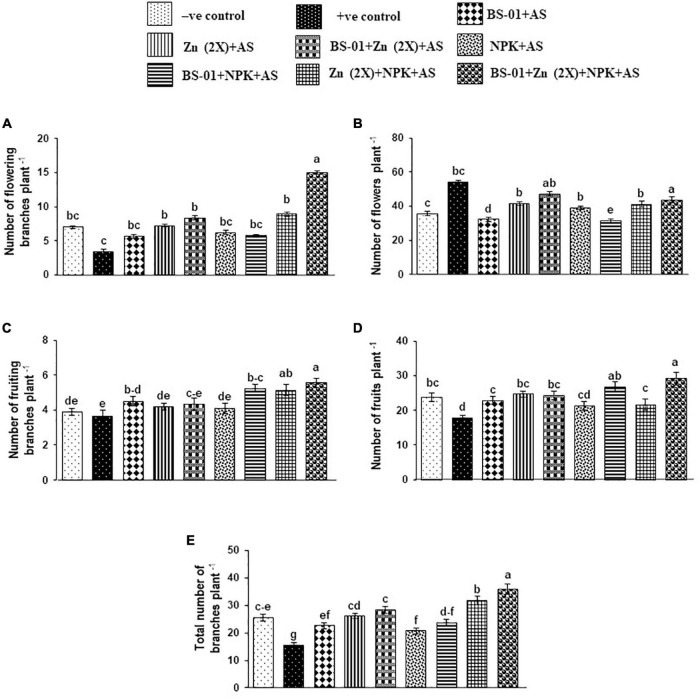
**(A–E)** Effect of *Bacillus subtilis* (BS-01) and mineral nutrients [Zn (2X: 10 kg acre^–1^) and NPK (64:46:50 kg acre^–1^)] on the number of flowering branches **(A)**, number of flowers **(B)**, number of fruiting branches **(C)**, number of fruits **(D)**, and the total number of branches **(E)** of tomato plants inoculated with *Alternaria solani* (AS) in the field after 30 days of pathogen inoculation (DPI). Values with different letters show a significant difference (*p* ≤ 0.05) in the mean value of replicates as determined by the LSD test. Error bars indicate standard errors of the mean of replicates. −ve control, without inoculation of AS and nutrient application; +ve control, with inoculation of AS only (foliar application).

Agronomic traits of the tomato plants, *viz*., the total number of branches (16 plant^–1^) and the number of fruits (NFR: 18 plant^–1^), were significantly decreased by 39% and 25% (*p* ≤ 0.05), respectively, in the positive control (plants inoculated with pathogen only), as compared to the negative control (plants free from pathogen and fertilizers), whereas the number of flowering branches (NFB: 3 plant^–1^), number of flowers (NF: 54 plant^–1^), and number of fruiting branches (NFRB: 4 plant^–1^) did not affect significantly (*p* ≥ 0.05) under EB stress in the positive control treatment. When BS-01 was applied in combination with Zn (2X) and NPK on the pathogen-infected plants, all said attributes were significantly greater (*p* ≤ 0.05) as compared to the positive and over negative controls. In a combination treatment of BS-01 + Zn (2X) + NPK, agronomic traits improved significantly by 125% (branches plant^–1^), 341% (flowering branches plant^–1^), 20% (flowers plant^–1^), 52% (fruiting branches plant^–1^), and 64% (fruits plant^–1^) as compared to the positive control. Likewise, these agronomic traits of this treatment [BS-01 + Zn (2X) + NPK] were significantly increased by 20–50% (*p* ≤ 0.05) over the negative control (without AS and fertilizer application), excluding the number of flowering branches plant^–1^, which were greater by 114%. The rest of the treatments exhibited a variable effect on the investigated agronomic traits of the tomato plant. Generally, the application of different treatments displayed significant improvement (*p* ≤ 0.05) in the total number of branches plant^–1^ and the number of fruits plant^–1^ (Figure 3).

### Growth Assessment

**FIGURE 4:**
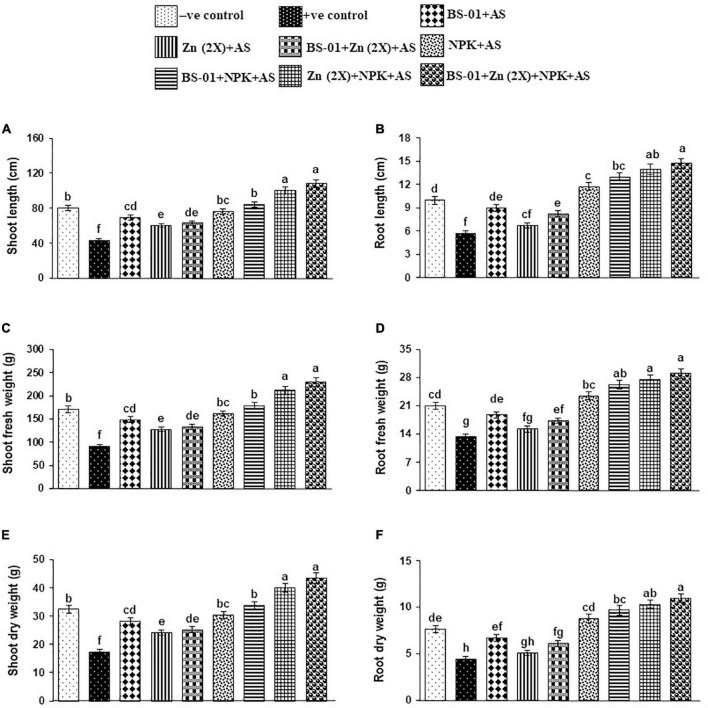
**(A–F)** Effect of *Bacillus subtilis* (BS-01) and mineral nutrients [Zn (2X: 10 kg acre^–1^) and NPK (64:46:50 kg acre^–1^)] on the shoot length **(A)**, shoot fresh weight **(B)** and shoot dry weight **(C)**, root length **(D)**, root fresh weight **(E)**, and root dry weight **(F)** of tomato plants inoculated with *Alternaria solani* (AS) in the field after 60 days of pathogen inoculation (DPI). Values with different letters show a significant difference (*p* ≤ 0.05) in the mean value of replicates as determined by the LSD test. Error bars indicate standard errors of the mean of replicates (*N* = 2). −ve control, without inoculation of AS and nutrient application; +ve control, with inoculation of AS only (foliar application).

Infected plants of positive control treatment resulted in a significant reduction in the length, and fresh and dry weight of shoots and roots by 36–47% (*p* ≤ 0.05) as compared to the healthy tomato plants in the negative control treatment. The effect of all treatments was significant on growth attributes over the positive control. When a combination of BS-01 + Zn (2X) + NPK was given to the infected plants, the significantly highest improvement of 100–160% (*p* ≤ 0.05) in the growth attributes was recorded, followed by the combination treatment of Zn (2X) + NPK showing 100–140% enhancement in growth. However, a combination of BS-01 + NPK was also found effective in significantly enhancing growth attributes by 950%–126% (*p* ≤ 0.05). Whereas, BS-01, Zn (2X), and NPK when applied alone had less effect on the growth of tomato plants inoculated with AS than all other tested treatments (Figure 4).

### Fruit Yield and Quality

Tomatoes were graded as A (> 3.5 cm), B (3.5–2.5 cm), and C (< 2.0 cm) on the basis of diameter (Table 6). Plants in the negative control treatment (healthy uninoculated and treated plants) produced the highest percentage (66%) of “B”-grade tomatoes ranging 3.5–2.5 cm followed by “A”-grade (24%) and “C”-grade (10%) tomatoes. In the positive control treatment (AS-inoculated plants), the numbers of “C”-grade tomatoes were the maximum (74%) followed by “B”-grade (19%) and “A”-grade (7%) tomatoes. A combined treatment effect of BS-01 + Zn (2X) + NPK produced the highest “A”-grade (68%) and “B”-grade (23%) tomatoes, and the lowest “C”-grade (9%) tomatoes. The second-best treatment was BS-01 + NPK with 56% “A”-, 32% “B”-, and 12% “C”-grade tomatoes. This was closely followed by a combined treatment Zn (2X) + NPK (A: 52%, B: 35%, and C: 13%). Whereas, NPK alone and a combination of BS-01 + Zn (2X) showed 40% “A”-, 34% “B”-, and 15% “C”-grade tomatoes. BS-01 and Zn alone provided more quantity of “B”-grade tomatoes in the same range (55–66%) (Table 6).

**TABLE 6:** Effect of *Bacillus subtilis* (BS-01) and mineral nutrients [Zn (2X: 10 kg acre^–1^) and NPK (64:46:50 kg acre^–1^)] on the grading of tomato fruits from the plants inoculated with *Alternaria solani* (AS) in the field after 90 days of pathogen inoculation (DPI).

Treatments		Grading percentage of tomato		
		A	B	C
T_1_	−ve control	24^g^	66^a^	10^h^
T_2_	+ve control (AS)	07^i^	19^i^	74^a^
T_3_	BS-01 + AS	22^h^	63^b^	15^e^
T_4_	Zn (2X) + AS	27^f^	54^c^	19^c^
T_5_	BS-01 + Zn (2X) + AS	38^e^	44^d^	18^d^
T_6_	NPK + AS	44^d^	36^e^	20^b^
T_7_	BS-01 + NPK + AS	56^b^	32^g^	12^g^
T_8_	Zn (2X) + NPK + AS	52^c^	35^f^	13^f^
T_9_	BS-01 + Zn (2X) + NPK + AS	68^a^	23^h^	09^i^

*Values with different letters show a significant difference (p ≤ 0.05) in the mean value of replicates as determined by the LSD test. −ve control, without inoculation of AS and nutrient application; +ve control, with inoculation of AS only (foliar application).*

The marketable yield (ton acre^–1^) was estimated from the total yield, by excluding the fruits having a fresh weight of less than 70 g and those showing symptoms of EB or any type of deformation (Table 7). EB reduced total yield and marketable yield by 46 and 65%, respectively, in the positive control (AS-inoculated plants) as compared to the healthy plants of the negative control treatment (uninoculated and treated plants). A combined treatment (T_9_) BS-01 + Zn (2X) + NPK produced significantly the maximum tomato yield [(total yield: 23.61 tons acre^–1^) and (marketable yield: 21.61 tons acre^–1^)] (*p* ≤ 0.05) as compared to the positive and negative control treatments. Likewise, all other combination treatments (T_5_, T_7_, and T_8_), *viz*., BS-01 + Zn (2X), BS-01 + NPK, and Zn (2X) + NPK, were produced significantly good total yield (18–19 tons acre^–1^) and marketable yield (15–17 tons acre^–1^) (*p* ≤ 0.05) over the positive and negative control treatments. When BS-01, NPK, and Zn have applied alone, the investigated yield (total yield: 14–17 tons acre^–1^ and marketable yield: 10–13 tons acre^–1^) were improved significantly (*p* ≤ 0.05) as compared to the positive control but significantly lower as compared to negative control and rest of the other treatments (Table 7).

**TABLE 7:** Effect of *Bacillus subtilis* (BS-01) and mineral nutrients [Zn (2X: 10 kg acre^–1^) and NPK (64:46:50 kg acre^–1^)] on the marketable, unmarketable, and total yield (tons acre^–1^) of tomato plants inoculated with *Alternaria solani* (AS) in the field after 160 days of pathogen inoculation (DPI).

Treatments		Marketable yield	Unmarketable yield	Total tomato yield
		(tons acre^–1^)		
T_1_	−ve control	16.04 ± 1.04^b^	2.58 ± 0.14^d^	18.62 ± 1.24^bc^
T_2_	+ ve control (AS)	5.55 ± 0.31^f^	4.50 ± 0.27^a^	10.05 ± 0.62^e^
T_3_	BS-01 + AS	11.22 ± 0.69^de^	4.37 ± 0.26^ab^	15.59 ± 0.81^cd^
T_4_	Zn (2X) + AS	10.50 ± 0.58^e^	4.18 ± 0.29^ab^	14.68 ± 0.85^d^
T_5_	BS-01 + Zn (2X) + AS	14.78 ± 0.82^bc^	3.80 ± 0.25^bc^	18.58 ± 0.97^bc^
T_6_	NPK + AS	13.22 ± 0.73^cd^	3.94 ± 0.22^ad^	17.16 ± 0.90^b–d^
T_7_	BS-01 + NPK + AS	17.11 ± 1.05^b^	2.04 ± 0.12^d^	19.15 ± 1.00^b^
T_8_	Zn (2X) + NPK + AS	14.88 ± 0.97^bc^	3.23 ± 0.18^c^	18.11 ± 1.05^bc^
T_9_	BS-01 + Zn (2X) + NPK + AS	21.61 ± 1.20^a^	2.00 ± 0.11^d^	23.61 ± 1.45^a^

*Values with different superscript letters show a significant difference (p ≤ 0.05) in the mean value of replicates as determined by LSD test ± value indicates the standard error mean of replicates. −ve control, without inoculation of AS and nutrient application; +ve control, with inoculation of AS only (foliar application).*

### Economic Analysis

All treatments (T_3_–T_9_) significantly reduced EB (*p* ≤ 0.05) and resulted in the improvement of marketable yield (kg acre^–1^) and consequently increased B:C over positive control (T_2_). The greatest net return 361,363 PKR acre^–1^ with the highest B:C (12.76) occurred in combination treatment (T_9_) of BS-01 + Zn (2X) + NPK compared to the positive control (B:C = 4.53). Among the rest of the treatments, a bilateral combination of BS-01 (T_7_) along with the recommended dose of chemical fertilizers [NPK (64:46:50 kg acre^–1^)] also gave a good net profit of 281,217 PKR acre^–1^ with greater B:C (10.62), which was computed. Besides, EB disease management using biocontrol agent (BS-01) with a double dose of Zn (2X: 10 kg acre^–1^) and recommended dose of NPK (64:46:50 kg acre^–1^) [T_5_: BS-01 + Zn (2X) and T_8_: Zn (2X) + NPK] managed disease sustainably because it would generate more economic return, i.e., 242,442 and 241,256 PKR acre^–1^ with the varying B:C values of 10.43 and 9.40, respectively. Whereas, the treatments with the sole application of BS-01 (T_3_) and NPK (T_6_) also showed the same range of B:C (8.40–8.81) with the variable net return of 79,350 PKR acre^–1^ and 212,638 PKR acre^–1^, respectively. On the other hand, the minimum net return (166,787 PKR acre^–1^) with B:C value of 8.03 was recorded with a single application Zn (2X) in T_4_ as compared to the other EB managing treatments (Table 8).

**TABLE 8:** Effect of *Bacillus subtilis* (BS-01) and mineral nutrients [Zn (2X: 10 kg acre^–1^) and NPK (64:46:50 kg acre^–1^)] on the marketable tomato yield (kg acre^–1^), total revenue (TC), total cost (TC), net return (NR), and benefit-cost ratio (B:C) of tomato plants inoculated with *Alternaria solani* (AS) in the field.

Treatments		Marketable tomato yield (kg acre^–1^)	Total Revenue (PKR)	Total cost (PKR)	Net Return		(B:C)
					(PKR)	(USD)	
T_1_	−ve control	14,551^b^	291,025	22,222	268,803	1,720	13.10
T_2_	+ ve control	5,035^f^	100,698	22,222	78,476	502	4.53
T_3_	BS-01 + AS	1,0179^de^	203,572	24,222	179,350	1,148	8.40
T_4_	Zn (2X) + AS	9,525^e^	190,509	23,722	166,787	1,067	8.03
T_5_	BS-01 + Zn (2X) + AS	13,408^bc^	268,164	25,722	242,442	1,552	10.43
T_6_	NPK + AS	11,993^cd^	239,860	27,222	212,638	1,361	8.81
T_7_	BS-01 + NPK + AS	15,522^b^	310,439	29,222	281,217	1,800	10.62
T_8_	Zn (2X) + NPK + AS	13,499^bc^	269,978	28,722	241,256	1,544	9.40
T_9_	BS-01 + Zn (2X) + NPK + AS	19,604^a^	392,085	30,722	361,363	2,313	12.76

*Values with different superscript letters show a significant difference (p ≤ 0.05) in the mean value of yield obtained from replicates as determined by the LSD test. −ve control, without inoculation of AS and nutrient application; +ve control, with inoculation of AS only (foliar application).*

### Association analysis

#### Numerical Classification

**FIGURE 5:**
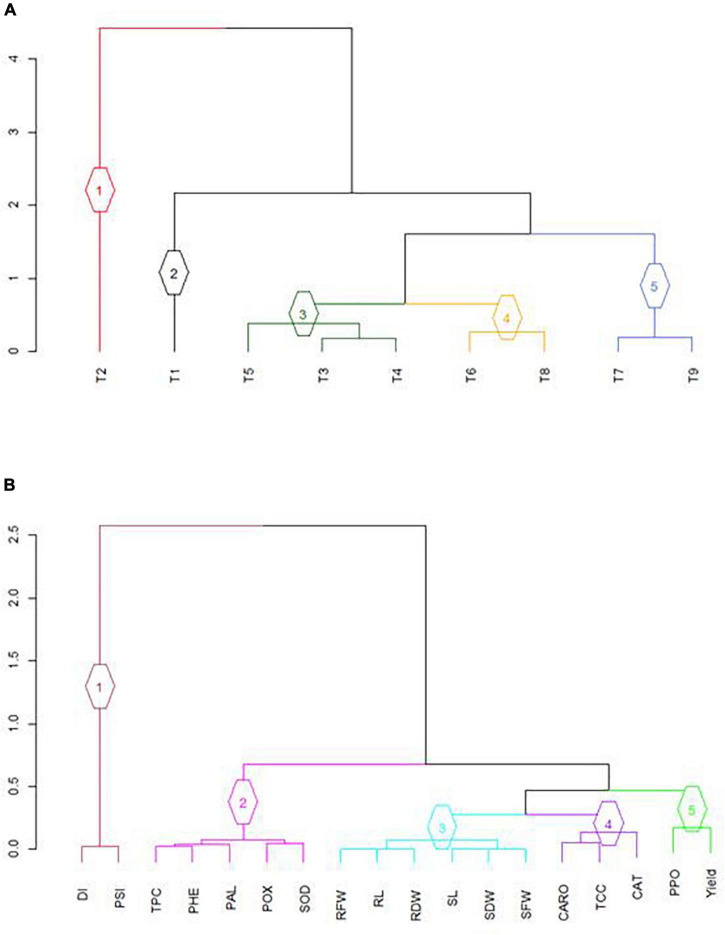
Optimized dendrogram computed from average squared Euclidean distance matrix using UPGMA clustering method: **(A)** classification based on nine treatments; and **(B)** classification based on disease, growth, physio-chemical, and yield attributes. Different groups are presented with different colors for easy group identification. T_1_, −ve control (without inoculation of AS); T_2_, +ve control (with AS inoculation); T_3_, BS-01 + AS; T_4_, Zn (2X) + AS; T_5_, BS-01 + Zn (2X) + AS; T_6_, NPK + AS; T_7_, BS-01 + NPK + AS; T_8_, Zn (2X) + NPK; and T_9_, BS-01 + Zn (2X) + NPK + AS. BS-01, *Bacillus subtilis*; AS, *Alternaria solani*; NPK, nitrogen, phosphorus, and potassium; Zn, Zinc.

The results of numerical classification (dendrogram) of different EB disease management treatments (T_3_–T_9_) according to their effects on physio-chemical, disease, growth, and yield attributes are depicted in Figure 5A. The nine treatments were classified into five clusters, and treatment/s in each cluster shared many characteristics. Cluster 1 was consisted of positive control (T_2_: *A. solani* only), whereas cluster 2 was composed of the negative control (T_1_: non-inoculated). Cluster 3 was represented by three treatments (T_3_: BS-01 + AS; T_4_: AS + Zn; and T_5_: BS-01 + AS + Zn), and cluster 4 included two treatments (T_6_: AS + NPK and T_8_: AS + NPK + Zn). At last, cluster 5 consisted of two treatments (T_7_: BS-01 + AS + NPK and T_9_: BS-01 + AS + Zn + NPK) against tomato EB. The major relationships between attributes (physio-chemical disease, growth, and yield attributes) under the effect of nine different treatments were represented in five partitions (Figure 5B). DI and PSI were more related to each other, therefore forming cluster 1. Different physio-chemical attributes (TPC, PHE, PAL, POX, and SOD) exhibited similar responses that were grouped in cluster 2. However, cluster 3 was a larger cluster represented by all growth attributes; cluster 4 comprised photosynthetic pigments (TCC and CARO) and CAT; and, finally, cluster 5 exhibited PPO and yield-related attributes (Figure 5B).

#### Classification Enhanced Biplot

**FIGURE 6:**
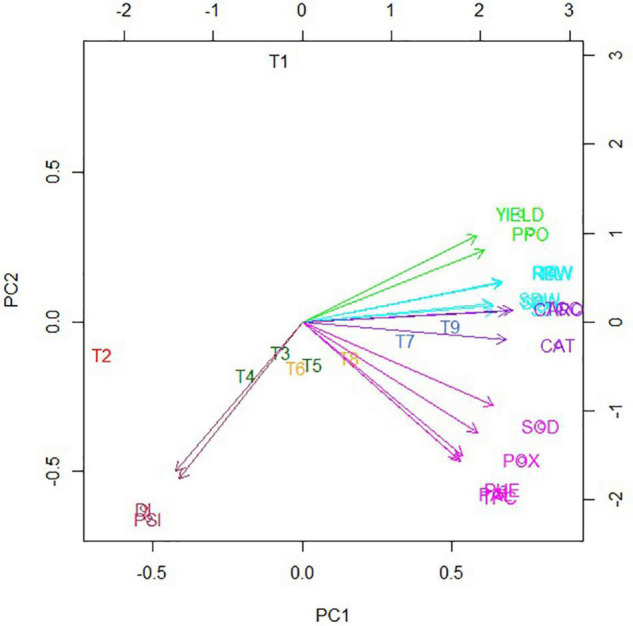
Biplot of the first two principal coordinates for nine treatments and collective data of disease, growth, physio-chemical, and yield attributes. The color scheme is kept the same as for respective dendrograms. DI, disease incidence; PSI, percent severity index; TCC, total chlorophyll content; CARO, carotenoids; TPC, total protein contact; PHE, total phenol content; SOD, superoxide dismutase; CAT, catalase; POX, peroxidase; PPO, polyphenol oxidase; PAL, phenylalanine ammonia-lyase; SL, shoot length; SFW, shoot fresh weight; SDW, shoot dry weight; RL, root length; RFW, root fresh weight; RDW, root dry weight. BS-01, *Bacillus subtilis*; AS, *Alternaria solani*; NPK, nitrogen, phosphorus, and potassium; Zn, Zinc. T_1_, −ve control (without inoculation of AS); T_2_, +ve control (with AS inoculation); T_3_, BS-01 + AS; T_4_, Zn (2X) + AS; T_5_, BS-01 + Zn (2X) + AS; T_6_, NPK + AS; T_7_, BS-01 + NPK + AS; T_8_, Zn (2X) + NPK; and T_9_, BS-01 + Zn (2X) + NPK + AS.

Classification enhanced biplot was constructed to visualize the effect of treatments on different assessed attributes. The color scheme for the classification of traits and treatments was kept the same to better understand the grouping (Figure 6). All growth, physio-chemical, and yield traits were grouped on the positive PC1 axis of the biplot, suggesting strong relationships among them. However, DI and PSI were occupied on the negative PC1 axis of the biplot strong, indicating strong relationships between them. It was also noticed T_3_, T_4_, T_5_, and T_6_ showed a similar effect on the different investigated traits thus clustered closer, whereas T_7_, T_8_, and T_9_ exhibited similar results to those assessed attributes are therefore clustered together. The closeness of the arrows of the said traits represents the relatedness of those traits. Disease attributes (DI and PSI) were grouped. The physio-chemical traits were in one group except for CAT and CARO. Growth attributes group in a separate cluster were behaving the same as CAT and CARO. Yield and PPO were in a separate group showing that PPO behaved differently than other parameters. Likewise, the closeness of the arrows and treatments represents their positive relationship. A perpendicular can be drawn from treatment over any of the mentioned attributes and compare those traits concerning a specific treatment. DI and PSI were higher in T_2_ (positive control), and they were close, too. The treatments were overlapping in some cases as well, i.e., treatments T_6_ and T_8_ were very close to the group containing treatments (T_3_, T_4_, and T_5_). Besides, treatments, *viz*., T_7_ and T_9_ in a group exhibited higher and positive values of growth attributes and closer as well.

#### Heatmap Visualization

**FIGURE 7:**
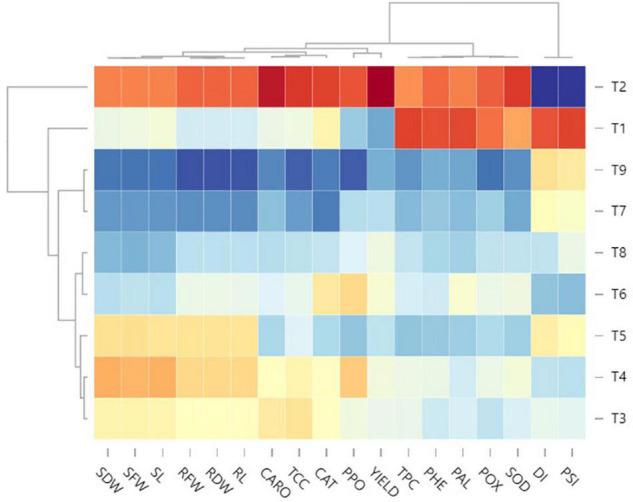
Standard optimized dendrograms and the associated ordered heatmap of standardized data of disease, growth, physio-chemical and yield attributes in nine treatments. BS-01, *Bacillus subtilis*; AS, *Alternaria solani*; NPK, nitrogen, phosphorus, and potassium; Zn, Zinc. T_1_, −ve control (without inoculation of AS); T_2_, +ve control (with AS inoculation); T_3_, BS-01 + AS; T_4_, Zn (2X) + AS; T_5_, BS-01 + Zn (2X) + AS; T_6_, NPK + AS; T_7_, BS-01 + NPK + AS; T_8_, Zn (2X) + NPK; and T_9_, BS-01 + Zn (2X) + NPK + AS.

To better decipher the complex interactions between EB and tomato yield under the disease managing nine treatments, a multivariate hierarchical cluster analysis was performed, and the variables were ordered by all attributes (physio-chemical, disease, growth, and yield) (Figure 7). Overall, high values of DI and PSI (intense blue color) in the positive control treatment were related to their adverse effect on the rest of the plant’s attributes (red color block). However, low DI and PSI (light blue to yellow) in other treatments were related to high values of growth, yield, and physio-chemical attributes. With the results of a heatmap, it was summarized that T_9_ followed by T_7_ exhibited highly significant potential to manage EB disease in the tomato plant with a tremendous enhancement in physio-chemical attributes (intense blue) that resulted in the maximum tomato yield.

## Discussion

### Fungal Load

The current study suggested that EB disease of tomato is effectively managed in a field trial with the application of biocontrol agent (BS-01) along with plant nutrients by varying degrees of resistance in plants. The biocontrol efficacy of *B. subtilis* (BS-01), Zn (2X: 10 kg acre^–1^), and NPK (recommended dose: 64:46:50 kg acre^–1^) either alone or in combination was verified and confirmed that an integrative effect of BS-01 + Zn (2X) + NPK against EB disease of tomato exhibited the highest reduction in fungal load (∼90%) followed by other two biological treatments, *viz*., BS-01 + Zn (2X) and BS-01 alone against EB. Likewise, Ongena et al. (2005) also reported that preventive measures such as pre-inoculation with *B. subtilis* sensitized tomato plants to react more efficiently to subsequent pathogen infection for effective disease control. Hence, plant protection as conferred by bacteria (*B. subtilis*) or nutrients used in this study could result from the induction of systemic resistance which enhances biological control over EB disease of tomato crop through direct antagonisms (Liu et al., 2018).

### Physio-Chemical Attributes

Different disease management treatments (T_3_–T_9_) were effectually modified health markers (photosynthetic pigments), stress markers (total phenolic content), and the activities of defensive enzymes (SOD, CAT, POX, PPO, and PAL) to a variable extent concerning the positive control. Synergistic cross-talk between the interactive effect of BS-01 (*B. subtilis*) and mineral nutrients (Zn and NPK) exhibited more significant enhancement in the said attributes as compared to their single effect. Health markers such as photosynthetic pigments (TCC and CARO) are the most important photosynthetic markers linked with the production aptitude (Torres et al., 2014). A significant reduction was detected in the positive control treatment (T_2_), which revealed the pathogen hijacked the pores to access the plant’s sugar for food, camouflaged the cell mechanism, and disrupted the production of photosynthetic pigments; therefore, the plants exhibited malfunctioning (Awan et al., 2019).

However, total phenolic content, TPC, and defense-related enzymes were not significantly increased in positive control as compared to the negative control (T_1_). Hence, the disease might shift the balance between oxidant/antioxidant statuses in favor of the oxidant chemicals through induction of transient, low-amplitude, and the first phase of reactive oxygen species (ROS) production (Awan et al., 2019; Shoaib et al., 2019). However, a significant elevation of PHE was observed in disease managing treatments, which could be further explained as a mechanism of defense, which may induce plant resistance through lignin biosynthesis to strengthen plant cell wall and induction of physical barrier against *A. solani* (Kaur et al., 2017).

Likewise, physio-chemical attributes were significantly enhanced after 10 and 30 DPI due to the effect of biocontrol and plant nutrients as compared to the positive control. ROS-scavenging systems then help to finely tune the ROS level under biotic stress through the over-production of key players (Torres, 2010). SOD enzyme accumulates H_2_O_2_ that diffuses into cells to act as an excellent antioxidant, and CAT is capable to scavenge H_2_O_2_ to mitigate the stress as well. Stimulation in POX activity might show oxidation of phenols, suberization, and lignification of host plant cells during the defense reaction against pathogenic agents (Elavarthi and Martin, 2010; Singh et al., 2016). High accumulation of PPO and PAL activities may reveal their involvement in phenol metabolism for providing the desired level of protection to tomato plants against *A. solani* (Awan et al., 2019). Generally, total phenolic content (PHE) and PAL activity increased tremendously at 30 DPI due to their role in SAR, suggesting their additive effect on salicylic acid, which may help the plant to systemically induce resistance against the pathogen after application of biocontrol and plant fertilizers (Awan et al., 2019; Shoaib et al., 2019). Production of total phenolics may induce the synthesis of host pathogenesis-related proteins (chitinase, 1,3-glucanase, or thaumatin), which likely to overcome the stress through limiting the reproduction and spread of the pathogen in infected tissue and also positively correlated with the plant resistance (Sels et al., 2008). Likewise, elevation in PAL activities may reveal their involvement in phenol metabolism for providing the desired level of protection to tomato plants against *A. solani*.

### Disease, Growth, and Agronomic Attributes

Disease management through BS-01 and mineral fertilization [Zn (2X) and NPK] could be regarded as an emerging approach that can improve the nutritional status of crops by managing EB disease. The results on growth (length and biomass of shoot and root), agronomy (total branches, flowering branches, fruiting branches, flowers, and fruits plant^–1^) and yield (quality and quantity) traits indicated that there was a significant difference among all the treatments. Trilateral interaction of BS-01 + Zn (2X) + NPK exhibited the highest reduction in disease (DI: 25%; PSI: 30%). However, bilateral interactions of BS-01 with fertilizers (Zn or NPK) also displayed a significant reduction in disease with similar DI: 30–40% and PSI: 35–38%. As a biocontrol agent, *B. subtilis* have been well known to promote mineralization, accumulation of the bioavailable forms of nutrients, nitrogen fixation, and increased root absorption ability, which might help in disease mitigation by providing important nutrients to the tomato plant as reported in former studies (Abde_Allaha et al., 2018; Masood et al., 2019). Besides, a strain of *B. subtilis* as a biocontrol agent is reported to enhance Zn solubilization in the rhizosphere of wheat and soybean, which resulted in higher crop growth due to the assimilation of Zn in seeds (Ramesh et al., 2014). The highest disease management due to bilateral and trilateral interaction of treatments might be associated with the ability of a biocontrol agent (BS-01) to the induction of systemic resistance against the pathogen (Bargaz et al., 2018). Zn might contribute to EB disease resistance by improving the structural integrity and permeability of cell membranes (Machado et al., 2018). Zn acts as a coenzyme in the biosynthesis of growth-promoting hormones (gibberellins and indole-3-acetic acid), for pigment biosynthesis, photosynthesis, respiration, cell wall development, and the rate of maturity function while acting as a structural and catalytic component of protein (Nguyen-Deroche et al., 2012; Hafeez et al., 2013). Zn may induce its toxic effect on *A. solani* by affecting its metabolism and enzymatic activity as reported earlier on other microorganisms by Zn nanoparticles. A balanced basal application of Zn was found to reduce the rice sheath blight severity (Ei Khaing et al., 2014), wheat root rot (Khoshgoftarmanesh et al., 2010), guar root rot (Wadhwa et al., 2014), and mung bean charcoal rot (Khan et al., 2018), because Zn can also suppress disease severity by acting as an antagonist for the veiling of active enzymatic groups in the fungus (Martos et al., 2016).

The results on growth (length and biomass of shoot and root), agronomic traits (total branches, flowering branches, fruiting branches, flowers, and fruits plant^–1^), and yield (quality and quantity) attributes indicated that there was a significant difference among all the treatments. Trilateral combination of BS-01 + Zn (2X) + NPK exhibited tremendous improvement up to threefold in the aforesaid attributes of the tomato crop as compared to the positive control and up to twofold as compared to the negative control. A bilateral combination of NPK either with BS-01 or Zn also resulted in greater improvement in the said plant characteristics of interest as compared to the positive control. The availability of essential plant nutrients in optimum quantity during the growth period would result in more vegetative growth (height and branches), thereby increasing plant biomass. The net results would be linked with enhancement in photosynthates, and their translocation toward the vegetative organs may have increased the longer period to the first fruit setting. Similar results were reported by Chatterjee et al. (2014) on the growth, phenological, and yield characters of tomatoes due to the combined application of inorganic and biofertilizers. The tomato plant needs NPK (macronutrients) in large amounts and Zn (micronutrient) in small amounts for growth and reproduction (Sainju et al., 2003). Moreover, NPK application increased vegetative growth and plant biomass that may signify the occurrence of excessive N. Besides, K plays a significant role in photosynthesis, plant respiration, transpiration, and nutrients translocation, whereas P is involved in energy transfer compounds, cell membranes, and phosphoproteins (Wissuwa, 2003). Adejumo (2010) reported that NPK fertilizers can control inflorescence blight in cashew and increase yield if apply appropriately. Similarly, Sten et al. (2017) also emphasized precision application and availability of NPK in managing fungal foliar diseases of potato, i.e., late blight, EB, and leaf blotch. The results proposed that nutrient imbalance and limited availability to plants might be consequences in response to *A. solani* infection as the pathogen can impair leaf cell wall, membrane permeability, and nutrient translocation (Huber et al., 2012).

### Economic Analysis

The ultimate aim of the grower is to secure maximum income out of the present resource. The current data showed that a trilateral combination [BS-01 + Zn (2X) + NPK] not only produced the maximum marketable yield (21.61 tons acre^–1^) but also provided the maximum net profit (Rs. 361,363 acre^–1^ or 2,313 USD) with the highest B:C (12.76) under sustainable crop management. Likewise, Anees et al. (2016) also reported that the application of nutrients including zinc increased the B:C by enhancing the grain yield of maize due to high fertilizer use efficiency. Moreover, Khan et al. (2018) reported that soil amendments with Zn and farmyard manure improved the grain yield of mungbean plants to profitable farming. Among the rest of the treatments, a combination of BS-01 along with the NPK (64:46:50 kg acre^–1^) also gave a good net return (Rs. 281,217 acre^–1^ or 1,800 USD) with good B:C (10.62). Similar results were obtained by Godwin et al. (2003) with the precision application of nitrogen fertilizers and gained the maximum profit in cereal production. Likewise, Farooqi et al. (2012) reported the positive impact of the foliar application of potassium on yield (B:C) and grain quality of maize. Hence, our study showed a biocontrol agent (BS-01) with a double dose of Zn (2X: 10 kg acre^–1^) and recommended dose of NPK (64:46:50 kg acre^–1^) proved effective and sustainable in EB disease management as it would generate more economic return.

## Conclusion

A net result acquired through the investigated attributes (physio-chemical assays, disease severity, agronomic traits, growth, and yield) indicated that the interactive application of *Bacillus subtilis* (BS-01) in combination with a double dose (2X) of Zn (10 kg acre^–1^) and recommended dose of NPK (64:46:50 acre^–1^) is an effective integrated approach for the management of tomato EB, which significantly alleviate the EB by exciting physio-chemical attributes in tomato plants and improved plant biological traits; hence, it will generate profitable tomato production with a good economic return.

## Data Availability Statement

The raw data supporting the conclusions of this article will be made available by the authors, without undue reservation.

## Author Contributions

AS and ZA did the conceptualization, performed the methodology, and investigated the data. ZA carried out the data curation and statistical analysis, wrote the original draft, and visualized the data. MI did the multivariate analysis. AS, BJ, and PA reviewed and edited the manuscript. AS supervised the data. BJ and PA carried out the funding acquisition. All authors contributed to the article and approved the submitted version.

## Conflict of Interest

The authors declare that the research was conducted in the absence of any commercial or financial relationships that could be construed as a potential conflict of interest.

## Publisher’s Note

All claims expressed in this article are solely those of the authors and do not necessarily represent those of their affiliated organizations, or those of the publisher, the editors and the reviewers. Any product that may be evaluated in this article, or claim that may be made by its manufacturer, is not guaranteed or endorsed by the publisher.

## References

[B1] Abde_Allaha E. F. Alqarawi A. A. Hashem A. Radhakrishnan R. Al-Huqail A. A. Al-Otibi F. O. N. (2018). Endophytic bacterium *Bacillus subtilis* (BERA 71) improves salt tolerance in chickpea plants by regulating the plant defense mechanisms. *J. Plant Interact.* 13 37–44. 10.1080/17429145.2017.1414321

[B2] Abiodun J. Osaretin B. I. Elizabeth T. A. Benson O. A. Ajibola P. A. (2017). Effectiveness of *Pseudomonas* species in the management of tomato early blight pathogen *Alternaria solani*. *Afr. J. Microbiol. Res.* 11 972–976. 10.5897/AJMR2017.8564

[B3] Adejumo T. O. (2010). Effect of NPK fertilization on yield and inflorescence blight of cashew (Anacardium occidentale). *J. Agric. Biotechol. Sustain. Dev.* 2 66–70.

[B4] Adhikari P. Oh Y. Panthee D. R. (2017). Current status of early blight resistance in tomato: an update. *Int. J. Mol. Sci.* 18:2019. 10.3390/ijms18102019 28934121 PMC5666701

[B5] Anees M. A. Ali A. Shakoor U. Ahmed F. Hasnain Z. Hussain A. (2016). Foliar Applied Potassium and Zinc Enhances Growth and Yield Performance of Maize under Rainfed Conditions. *Int. J. Agric. Biol.* 18 1025–1032. 10.17957/IJAB/15.0204 29653435

[B6] Awan Z. A. Shoaib A. (2019). Combating early blight infection by employing *Bacillus subtilis* in combination with plant fertilizers. *Curr. Plant Biol.* 20:100125. 10.1016/j.cpb.2019.100125

[B7] Awan Z. A. Shoaib A. Khan K. A. (2018). Variations in total phenolics and antioxidant enzymes cause phenotypic variability and differential resistant response in tomato genotypes against early blight disease. *Sci. Hortic.* 239 216–223. 10.1016/j.scienta.2018.05.044

[B8] Awan Z. A. Shoaib A. Khan K. A. (2019). Crosstalk of zn in combination with other fertilizers underpins interactive effects and induces resistance in tomato plant against early blight disease. *Plant Pathol. J.* 35 330–340. 10.5423/PPJ.OA.01.2019.0002 31481856 PMC6706011

[B9] Bargaz A. Lyamlouli K. Chtouki M. Zeroual Y. Dhiba D. (2018). Soil Microbial Resources for Improving Fertilizers Efficiency in an Integrated Plant Nutrient Management System. *Front. Microbiol.* 9:1606. 10.3389/fmicb.2018.01606 30108553 PMC6079243

[B10] Bauchet G. Grenier S. Samson N. Segura V. Kende A. Beekwilder J. (2017). Identification of major loci and genomic regions controlling acid and volatile content in tomato fruit: implications for flavor improvement. *New Phytol.* 215 624–641. 10.1111/nph.14615 28585324

[B11] Chatterjee R. Bandyopadhyay S. Jana J. C. (2014). Impact of Organic Amendments and Inorganic Fertilizers on Production Potential, Nitrogen Use Efficiency and Nitrogen Balance in Tomato (*Lycopersicon esculentum* Mill.). *Int. J. Sci. Res. Knowl.* 2 233–240. 10.12983/ijsrk-2014-p0233-0240

[B12] Decelis S. Sardella D. Triganza T. Brincat J. P. Gatt R. Valdramidis V. P. (2017). Assessing the anti-fungal efficiency of filters coated with zinc oxide nanoparticles. *R. Soc. Open Sci.* 4:161032. 10.1098/rsos.161032 28572995 PMC5451796

[B13] Dordas C. (2008). Role of nutrients in controlling plant diseases in sustainable agriculture. A review. *Agron. Sustain. Dev.* 28 33–46. 10.1051/agro:2007051

[B14] Ei Khaing E. Abidin Mior Ahmad Z. MuiYun W. Razi Ismail M. (2014). Effects of Silicon, Copper and Zinc Applications on Sheath Blight Disease Severity on Rice. *World J. Agric. Res.* 2 309–314. 10.12691/wjar-2-6-11

[B15] Elavarthi S. Martin B. (2010). Spectrophotometric assays for antioxidant enzymes in plants. *Methods Mol. Biol.* 639 273–281. 10.1007/978-1-60761-702-0_16 20387052

[B16] Farooqi M. Q. U. Ahmad R. Wariach E. A. Arfan M. (2012). Effect of supplemental foliar applied potassium on yield and grain quality of autumn planted maize (*Zea mays* L) under water stress. *J. Food Agric. Vet. Sci.* 2 8–12.

[B17] Godwin R. J. Wood G. A. Taylor J. C. Knight S. M. Welsh J. P. (2003). Precision Farming of Cereal Crops: a Review of a Six Year Experiment to develop Management Guidelines. *Biosyst. Eng.* 84 375–391. 10.1016/S1537-5110(03)00031-X

[B18] Hafeez B. Khanif Y. M. Saleem M. (2013). Role of Zinc in Plant Nutrition- A Review. *Am. J. Exp. Agric.* 3 374–391.

[B19] Hasanuzzaman M. Bhuyan M. Nahar K. Hossain M. Mahmud J. Hossen M. (2018). Potassium: a Vital Regulator of Plant Responses and Tolerance to Abiotic Stresses. *Agronomy* 8:31. 10.3390/agronomy8030031

[B20] Huber D. Römheld V. Weinmann M. (2012). “Relationship between Nutrition, Plant Diseases and Pests,” in *Marschner’s Mineral Nutrition of Higher Plants*, ed. Marschner P. (Amsterdam: Elsevier), 283–298. 10.1016/B978-0-12-384905-2.00010-8

[B21] Imran M. Ali A. Ashfaq M. Hassan S. Culas R. Ma C. (2018). Impact of Climate Smart Agriculture (CSA) Practices on Cotton Production and Livelihood of Farmers in Punjab, Pakistan. *Sustainability* 10:2101. 10.3390/su10062101

[B22] Kaur H. Salh P. K. Singh B. (2017). Role of defense enzymes and phenolics in resistance of wheat crop (*Triticum aestivum* L.) towards aphid complex. *J. Plant Interact.* 12 304–311. 10.1080/17429145.2017.1353653

[B23] Khan K. A. Shoaib A. Arshad Awan Z. Basit A. Hussain M. (2018). *Macrophomina phaseolina* alters the biochemical pathway in *Vigna radiata* chastened by Zn 2+ and FYM to improve plant growth. *J. Plant Interact.* 13 131–140. 10.1080/17429145.2018.1441451

[B24] Khoshgoftarmanesh A. H. Kabiri S. Shariatmadari H. Sharifnabi B. Schulin R. (2010). Zinc nutrition effect on the tolerance of wheat genotypes to Fusarium root-rot disease in a solution culture experiment. *Soil Sci. Plant Nutr.* 56 234–243. 10.1111/j.1747-0765.2009.00441.x

[B25] Liu H. Ren L. Spiertz H. Zhu Y. Xie G. H. (2015). An economic analysis of sweet sorghum cultivation for ethanol production in North China. *Glob. Change Biol. Bioenergy* 7 1176–1184. 10.1111/gcbb.12222

[B26] Liu K. Mcinroy J. A. Hu C. Kloepper J. W. Pathology P. (2018). Mixtures of plant-growth-promoting rhizobacteria enhance biological control of multiple plant diseases and plant-growth promotion in the presence of pathogens. *Plant Dis*. 102, 67–72. 30673446 10.1094/PDIS-04-17-0478-RE

[B27] Machado P. P. Steiner F. Zuffo A. M. Machado R. A. (2018). Could the Supply of Boron and Zinc Improve Resistance of Potato to Early Blight?. *Potato Res.* 61 169–182. 10.1007/s11540-018-9365-4

[B28] Martos S. Gallego B. Cabot C. Llugany M. Barceló J. Poschenrieder C. (2016). Zinc triggers signaling mechanisms and defense responses promoting resistance to *Alternaria brassicicola* in *Arabidopsis thaliana*. *Plant Sci.* 249 13–24. 10.1016/j.plantsci.2016.05.001 27297986

[B29] Masood S. Zhao X. Q. Shen R. F. (2019). Bacillus pumilus increases boron uptake and inhibits rapeseed growth under boron supply irrespective of phosphorus fertilization. *AoB Plants* 11:plz036. 10.1093/aobpla/plz036 31321016 PMC6626985

[B30] Meitei K. M. Bora G. C. Borah P. K. (2014). Screening of Tomato Genotypes for Resistance to Early Blight (*Alternaria solani*). *Int. J. Sci. Res.* 3 384–387.

[B31] Nashwa S. M. A. Abo-elyousr K. A. M. (2012). Evaluation of Various Plant Extracts against the Early Blight Disease of Tomato Plants under Greenhouse and Field Conditions. *Plant Pathol.* 48 74–79.

[B32] Nguyen-Deroche T. L. N. Caruso A. Le T. T. Bui T. V. Schoefs B. Tremblin G. (2012). Zinc affects differently growth, photosynthesis, antioxidant enzyme activities and phytochelatin synthase expression of four marine diatoms. *ScientificWorldJournal* 2012:982957. 10.1100/2012/982957 22645501 PMC3356767

[B33] Ongena M. Duby F. Jourdan E. Beaudry T. Jadin V. Dommes J. (2005). Bacillus subtilis M4 decreases plant susceptibility towards fungal pathogens by increasing host resistance associated with differential gene expression. *Appl. Microbiol. Biotechnol.* 67 692–698. 10.1007/s00253-004-1741-0 15578181

[B34] Pandey K. K. Pandey P. K. Kalloo G. Banerjee M. K. (2003). Resistance to early blight of tomato with respect to various parameters of disease epidemics. *J. Gen. Plant Pathol.* 69 364–371. 10.1007/s10327-003-0074-7

[B35] Pervaiz U. Salam A. Jan D. Khan A. Iqbal M. (2018). Adoption Constraints of Improved Technologies Regarding Tomato Cultivation in District Mardan, KP. *Sarhad J. Agric.* 34 428–434. 10.17582/journal.sja/2018/34.2.428.434

[B36] Ramesh A. Sharma S. K. Sharma M. P. Yadav N. Joshi O. P. (2014). Inoculation of zinc solubilizing *Bacillus aryabhattai* strains for improved growth, mobilization and biofortification of zinc in soybean and wheat cultivated in Vertisols of central India. *Appl. Soil Ecol.* 73 87–96. 10.1016/j.apsoil.2013.08.009

[B37] Sainju U. M. Dris R. Singh B. (2003). Mineral nutrition of tomato. *J. Food Agric. Environ.* 1 176–183.

[B38] Sels J. Mathys J. De Coninck B. M. A. Cammue B. P. A. De Bolle M. F. C. (2008). Plant pathogenesis-related (PR) proteins: a focus on PR peptides. *Plant Physiol. Biochem.* 46 941–950. 10.1016/j.plaphy.2008.06.011 18674922

[B39] Shoaib A. Awan Z. A. (2020). Mineral fertilizers improve defense related responses and reduce early blight disease in tomato (*Solanum lycopersicum* L.). *J. Plant Pathol.* 103 217–229.

[B40] Shoaib A. Awan Z. A. Khan K. A. (2019). Intervention of antagonistic bacteria as a potential inducer of disease resistance in tomato to mitigate early blight. *Sci. Hortic.* 252 20–28. 10.1016/j.scienta.2019.02.073

[B41] Singh U. B. Malviya D. Wasiullah, Singh S. Pradhan J. K. Singh B. P. (2016). Bio-protective microbial agents from rhizosphere eco-systems trigger plant defense responses provide protection against sheath blight disease in rice (*Oryza sativa* L.). *Microbiol. Res.* 192 300–312. 10.1016/j.micres.2016.08.007 27664749

[B42] Sten J. Chandra B. Viswavidyalaya K. Mahapatra S. Chandra B. Viswavidyalaya K. (2017). Effect of different levels of NPK on foliar diseases of potato under different fertility gradient soil in field Effect of different levels of NPK on foliar diseases of potato under different fertility gradient soil in field. *J. Crop Weed* 13 151–156.

[B43] Syed-Ab-Rahman S. F. Carvalhais L. C. Chua E. Xiao Y. Wass T. J. Schenk P. M. (2018). Identification of Soil Bacterial Isolates Suppressing Different Phytophthora spp. and Promoting Plant Growth. *Front. Plant Sci.* 9:1502. 10.3389/fpls.2018.01502 30405657 PMC6201231

[B44] Syed-Ab-Rahman S. F. Xiao Y. Carvalhais L. C. Ferguson B. J. Schenk P. M. (2019). Suppression of *Phytophthora capsici* infection and promotion of tomato growth by soil bacteria. *Rhizosphere* 9 72–75. 10.1016/j.rhisph.2018.11.007

[B45] Torres M. A. (2010). ROS in biotic interactions. *Physiol. Plant.* 138 414–429. 10.1111/j.1399-3054.2009.01326.x 20002601

[B46] Torres P. B. Chow F. Furlan C. M. Mandelli F. Mercadante A. dos Santos D. Y. A. C. (2014). Standardization of a protocol to extract and analyze chlorophyll a and carotenoids in *Gracilaria tenuistipitata* var. liui. zhang and xia (rhodophyta). *Braz. J. Oceanogr.* 62 57–63. 10.1590/S1679-87592014068106201

[B47] Wadhwa N. Joshi U. N. Mehta N. (2014). Zinc Induced Enzymatic Defense Mechanisms in Rhizoctonia Root Rot Infected Clusterbean Seedlings. *J. Bot.* 2014 1–7. 10.1155/2014/735760

[B48] Wissuwa M. (2003). How Do Plants Achieve Tolerance to Phosphorus Deficiency? Small Causes with Big Effects. *Plant Physiol.* 133 1947–1958. 10.1104/pp.103.029306 14605228 PMC300746

